# Diagnostic value of kappa free light chain index in patients with primary progressive multiple sclerosis – a multicentre study

**DOI:** 10.3389/fimmu.2023.1327947

**Published:** 2023-12-19

**Authors:** Harald Hegen, Klaus Berek, Paola Cavalla, Mikael Christiansen, Andreja Emeršič, Massimiliano Di Filippo, Lorenzo Gaetani, Michaela Hassler, Cyra Leurs, Dejan Milosavljevic, Vincent van Pesch, Thor Petersen, Stefan Presslauer, Igal Rosenstein, Uroš Rot, Christine Schnabl, Charlotte Teunissen, Domizia Vecchio, Marco Vercellino, Florian Deisenhammer

**Affiliations:** ^1^ Department of Neurology, Medical University of Innsbruck, Innsbruck, Austria; ^2^ Multiple Sclerosis Center and Neurologia I U, Department of Neuroscience and Mental Health, AOU Città della Salute e della Scienza di Torino, Torino, Italy; ^3^ Department of Clinical Biochemistry, Regional Hospital in Horsens, Horsens, Denmark; ^4^ Department of Neurology, University Medical Centre Ljubljana, Ljubljana, Slovenia; ^5^ Section of Neurology, Department of Medicine and Surgery, University of Perugia, Perugia, Italy; ^6^ FH Campus Wien, University of Applied Sciences, Vienna, Austria; ^7^ Department of Neurology and Neurophysiology, Amsterdam Neuroscience, Amsterdam UMC, Location VUMC, Amsterdam, Netherlands; ^8^ Cliniques Universitaires Saint-Luc, UCLouvain, Brussels, Belgium; ^9^ Sygehus Sønderjylland, Department of Regional Health Research, University Hospital of Southern Denmark, Hadersleben, Denmark; ^10^ Department of Neurology, Klinikum Ottakring, Vienna, Austria; ^11^ Department of Clinical Neuroscience, Institute of Neuroscience and Physiology at Sahlgrenska Academy, University of Gothenburg, Gothenburg, Sweden; ^12^ Faculty of Medicine, University of Ljubljana, Ljubljana, Slovenia; ^13^ Neurochemistry Laboratory, Department of Clinical Chemistry, Amsterdam UMC, Location Vrije Universiteit, Amsterdam Neuroscience, Amsterdam, Netherlands; ^14^ Neurology Unit Department of Translational Medicine, Maggiore della Carità University Hospital, Novara, Italy

**Keywords:** cerebrospinal fluid, kappa free light chain, primary progressive, multiple sclerosis, diagnosis, sensitivity

## Abstract

**Background:**

Kappa free light chains (κ-FLC) in the cerebrospinal fluid (CSF) are an emerging biomarker in multiple sclerosis (MS).

**Objective:**

To investigate whether κ-FLC index has similar diagnostic value in patients with primary progressive multiple sclerosis (PPMS) compared to oligoclonal bands (OCB).

**Methods:**

Patients with PPMS were recruited through 11 MS centres across 7 countries. κ-FLC were measured by immunonephelometry/-turbidimetry. OCB were determined by isoelectric focusing and immunofixation.

**Results:**

A total of 174 patients (mean age of 52±11 years, 51% males) were included. κ-FLC index using a cut-off of 6.1 was positive in 161 (93%) and OCB in 153 (88%) patients.

**Conclusion:**

κ-FLC index shows similar diagnostic sensitivity than OCB in PPMS.

## Introduction

Diagnosis of multiple sclerosis (MS) requires the combination of clinical signs and symptoms with paraclinical findings obtained by magnetic resonance imaging and cerebrospinal fluid (CSF) analysis (1). Evidence of intrathecal immunoglobulin G (IgG) synthesis in the CSF, although not specific for MS (2), substitutes for dissemination in time according to current diagnostic criteria and increases diagnostic certainty (1). The gold standard to determine intrathecal IgG synthesis is the detection of CSF-restricted oligoclonal bands (OCB) (3). However, advances in laboratory methods brought up κ-free light chains (FLC) as new CSF biomarker, which are produced in excess over intact immunoglobulins and also accumulate in CSF in case of central nervous system-derived inflammation (4). A large body of evidence showed high diagnostic accuracy of κ-FLC index in MS with sensitivity and specificity of approximately 90% similar to OCB (5). Studies were largely confined to patients with relapsing-remitting MS, i.e. studies on the diagnostic value of κ-FLC index in patients with primary progressive MS (PPMS) are still lacking, which is why we performed the present work.

## Methods

From a recent meta-analysis (5), we (HH, KB) identified those studies which included patients with PPMS (n=11), contacted the first and/or corresponding authors and invited them to contribute source data of PPMS patients for the present analysis. The following data were requested: age, sex, diagnosis, applied diagnostic criteria, OCB results (positive/negative), albumin quotient (Q_alb_), CSF and serum κ-FLC concentration, κ-FLC index, laboratory method used for κ-FLC detection and, if available, further CSF results (red blood and white blood cell (WBC) count, Q_IgG_, IgG index).

### κ-FLC assay and calculation of intrathecal κ-FLC synthesis

κ-FLC concentrations in CSF and serum were determined by each centre as specified in the previous publications (5) either by nephelometry or turbidimetry using a serum κ-FLC immunoassay (N Latex [Siemens] or Freelite [Binding Site]) according to the manufacturers’ instructions (6, 7).

Intrathecal synthesis of κ-FLC was determined by following formula considering serum κ-FLC concentrations and blood-CSF-barrier function.


$$\kappa -FLC\;index\;=\;\frac{\kappa -FLC_{CSF}\;/\;\kappa -FLC_{Serum}}{Albumin_{CSF}\;/\;Albumin_{Serum}}$$

A κ-FLC index >6.1 denoted presence of intrathecal κ-FLC synthesis (termed ‘positive’), a κ-FLC index ≤6.1 denoted absence of intrathecal synthesis (termed ‘negative’) (5).

### Statistics

Statistical analysis was performed by SPSS 27.0 (SPSS Inc, Chicago, IL, USA). Data are displayed as number (frequencies) and median (interquartile range), as appropriate. Group comparisons of categorical variables were done by χ^2^ test and of metric variables by Mann-Whitney-U test. Correlation analyses were performed using Spearman correlation coefficient (r). Concordance between dichotomous results, e.g. of κ-FLC index and OCB, was calculated as the number of positives revealed by both methods and the number of negatives revealed by both methods, related to the total number of patients, and expressed as percentage. Agreement between dichotomous results of κ-FLC index and OCB was also assessed by the adjusted kappa statistics, which considers the unequal distribution of OCB positive and negative test results in our cohort (as we included only PPMS patients) (8). A two-tailed p-value<0.05 was considered statistically significant.

## Results

**Figure 1:**
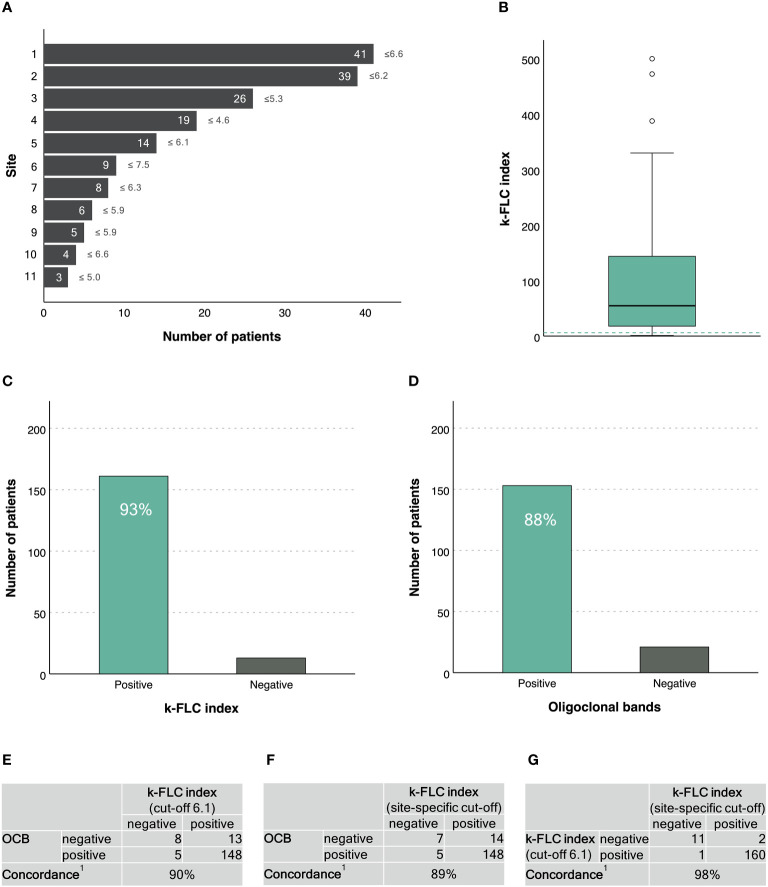
κ-FLC index in patients with primary progressive MS. **(A)** Number of patients (and κ-FLC index cut-off points) provided per MS centre^2^. **(B)** Distribution of κ-FLC index^3^. **(C)** Prevalence of positive/negative κ-FLC index (using a cut-off of 6.1). **(D)** Prevalence of positive/negative OCB. **(E)** Agreement between κ-FLC index (using a cut-off of 6.1) and OCB. **(F)** Agreement between κ-FLC index (using the centre-specific cut-offs) and OCB. **(G)** Agreement between κ-FLC index using a cut-off of 6.1 or centre-specific cut-offs. ^1^ Concordance was calculated as the number of positives revealed by both methods and the number of negatives revealed by both methods, related to the total number of patients, and expressed as percentage. ^2^ Patients’ data were drawn out of following publications and/or recruited through following MS centres: 1 Leurs et al. Mult Scler 2020;26(8):912-923. 2 Cavalla et al. J Neuroimmunol 2020;339:577122 (n=11) and unpublished data of patients from AOU Città della Salute e della Scienza di Torino, Torino, Italy (n=28). 3 Emersic et al. Clin Chim Acta 2019;489:109-116 (n=8) and unpublished data of patients from University Medical Centre Ljubljana, Ljubljana, Slovenia (n=18). 4 Rosenstein et al. J Neurochem 2021;159(3):618-628. 5 Unpublished data of patients from Medical University of Innsbruck, Innsbruck, Austria. 6 Christiansen et al. Clin Chem Lab Med 2018;57(2):210-220. 7 Bayart et al. Acta Neurol Scand 2018;138(4):352-358. 8 Presslauer et al. Mult Scler 2016;22(4):502-10. 9 Presslauer et al. J Neurol 2008;255(10):1508-14. 10 Gaetani et al. J Neuroimmunol 2020;339:577108. 11 Vecchio et al. Sci Rep 2020;10(1):20329. ^3^ The horizontal, green, dashed line indicates a κ-FLC index at 6.1. FLC, free light chain; OCB, oligoclonal bands.

A total of 174 PPMS patients with a mean age of 52±11 years and a balanced sex ratio (51% males) were recruited through 11 MS centres across 7 countries (**Figure 1A**). Demographics, main clinical characteristics and CSF findings are given in **Table 1**.

**Table 1:** Demographics, clinical characteristics and CSF findings.

Number of patients	174
Demographics	
Age (years), mean±SD	52±11
Sex (male), n (%)	88 (51)
Clinical characteristics	
Diagnoses made according to McDonald criteria, n (%) 2005 2010 2017	19 (11) 59 (34) 96 (55)
Cerebrospinal fluid findings	
RBC count (/μl)	0 (0-1)
WBC count (/μl)	3 (2-6)
Albumin quotient	5.3 (4.2-7.2)
IgG index	0.72 (0.58-1.05)
Oligoclonal IgG bands^1^, n (%)	153 (88)
CSF κ-FLC (mg/l)	4.58 (1.46-10.20)
Serum κ-FLC (mg/l)	14.18 (10.18-17.71)
Centre-specific differences	
Platform Nephelometry, n (%) Turbidimetry, n (%)	116 (67) 58 (33)
Immunoassay N Latex, n (%) Freelite, n (%)	100 (58) 73 (42)

Data are shown as median and interquartile range unless specified otherwise.

^1^ OCB positivity was defined as ≥2 CSF-restricted bands in 7 studies (including 113 patients), ≥3 CSF-restricted bands in 3 studies (including 20 patients), while one study (including 41 patients) did not specify the used cut-off.

CSF, cerebrospinal fluid; FLC, free light chain; IgG, immunoglobulin G; RBC, red blood cell; SD, standard deviation; WBC, white blood cell.

κ-FLC index had a median of 54.6 (IQR 17.8-144.0) (**Figure 1B**). κ-FLC index did not statistically significantly differ between males and females (p=0.151), did not correlate with patients’ age (r=-0.123, p=0.106), but significantly correlated with CSF WBC (r=0.23, p<0.023) and IgG index (r=0.80, p<0.001).

Using a cut-off of 6.1, κ-FLC index was denoted positive in 161 (93%) patients (**Figure 1C**). OCB were positive in 153 (88%) patients (**Figure 1D**). Concordance between κ-FLC index and OCB was reached in 90% of cases (**Figure 1E**). The adjusted kappa statistics revealed a substantial agreement (0.79).

The different MS centres used different cut-off points for κ-FLC index to denote a positive result ranging from 4.6 to 7.5 (**Figure 1A**). Applying site-specific cut-off points also revealed a (median) diagnostic sensitivity for κ-FLC index of 93%. The concordance with OCB was 89% (**Figure 1F**), and the adjusted kappa statistics showed a substantial agreement (0.78).

The agreement between κ-FLC index using site-specific cut-off points or a fixed cut-off at 6.1 reached 98% (**Figure 1G**). Neither the type of platform (nephelometry vs. turbidimetry) nor the type of immunoassay (N Latex vs. Freelite) did impact on diagnostic sensitivity (χ^2^, p<0.415 and 0.777).

## Discussion

In this study, we demonstrated that κ-FLC index reaches a diagnostic sensitivity of 93% in patients with PPMS which is similar to OCB. Even though a multitude of studies have already reported a comparable diagnostic accuracy of κ-FLC index and OCB (5), these studies were largely confined to patients with a relapsing-remitting disease course. Providing evidence on the performance of κ-FLC index in PPMS is of high clinical importance, as it has been suggested to include intrathecal κ-FLC synthesis into the next revision of MS diagnostic criteria (9).

We observed that there was no difference in diagnostic sensitivity of κ-FLC index whether a fixed cut-off of 6.1 –as retrieved by a recent meta-analysis (5)– or each the site-specific cut-off points were used. This might be due to the fact that the site-specific cut-off points were close to 6.1 (ranging from 4.6 to 7.5) and that the vast majority of MS patients shows considerably higher κ-FLC indices (approximately 95% of patients with positive κ-FLC index had values of 10 or higher).

We did not observe any impact of type of platform or assay on κ-FLC index positivity. This is in line with two prior large analyses (5, 10) and might be explained by a smaller laboratory variation due to using the CSF/serum κ-FLC ratio within the index rather than absolute concentrations. Furthermore, we compared the positivity rate of κ-FLC index and as the majority of patients show considerably elevated κ-FLC index values, a certain variation does not change classification in positive or negative.

There are some limitations of the study. This is a retrospective study with all the inherent limitations of such a design. The analytic performance of OCB detection probably differed between centres, as different methods (e.g. commercial versus in-house assays) were applied; and the interpretation of results is rater-dependent (4). It cannot be excluded that these differences might have influenced diagnostic sensitivity of OCB. We would like to state that one of the advantages of κ-FLC is the reliable and rater-independent determination which should overcome technical difficulties of OCB.

Altogether, we provide profound evidence that κ-FLC index and OCB show similar diagnostic performance in patients with PPMS and, thereby, filled the prior scientific and practical gap in this field. Determination of κ-FLC index is a fast, cost-effective and – as already mentioned above – a rater-independent method. By showing high diagnostic performance independent of the initial MS disease courses, it might serve as alternative to OCB testing in patients with suspected MS (5, 8).

## Data availability statement

The raw data supporting the conclusions of this article will be made available by the authors, without undue reservation.

## Ethics statement

The studies involving humans were approved by local ethic committees of participating centres. The studies were conducted in accordance with the local legislation and institutional requirements. The human samples used in this study were acquired as part of previous studies for which ethical approval was obtained. Written informed consent for participation was not required from the participants or the participants’ legal guardians/next of kin in accordance with the national legislation and institutional requirements.

## Author contributions

HH: Conceptualization, Data curation, Formal analysis, Writing – original draft. KB: Data curation, Writing – review & editing. PC: Data curation, Writing – review & editing. MC: Data curation, Writing – review & editing. AE: Data curation, Writing – review & editing. MD: Data curation, Writing – review & editing. LG: Data curation, Writing – review & editing. MH: Data curation, Writing – review & editing. CL: Data curation, Writing – review & editing. DM: Data curation, Writing – review & editing. Vv: Data curation, Writing – review & editing. TP: Data curation, Writing – review & editing. SP: Data curation, Writing – review & editing. IR: Data curation, Writing – review & editing. UR: Data curation, Writing – review & editing. CS: Data curation, Writing – review & editing. CT: Data curation, Writing – review & editing. DV: Data curation, Writing – review & editing. MV: Conceptualization, Writing – review & editing. FD: Writing – review & editing.

## References

[1] Thompson AJ Banwell BL Barkhof F Carroll WM Coetzee T Comi G. Diagnosis of multiple sclerosis: 2017 revisions of the McDonald criteria. Lancet Neurol (2018) 17(2):162–73. doi: 10.1016/S1474-4422(17)30470-2 29275977

[2] Deisenhammer F Bartos A Egg R Gilhus NE Giovannoni G Rauer S. Guidelines on routine cerebrospinal fluid analysis. Rep an EFNS task force Eur J Neurol (2006) 13(9):913–22. 10.1111/j.1468-1331.2006.01493.x 16930354

[3] Freedman MS Thompson EJ Deisenhammer F Giovannoni G Grimsley G Keir G. Recommended standard of cerebrospinal fluid analysis in the diagnosis of multiple sclerosis: a consensus statement. Arch Neurol (2005) 62(6):865–70. doi: 10.1001/archneur.62.6.865 15956157

[4] Hegen H Berek K Deisenhammer F. Cerebrospinal fluid kappa free light chains as biomarker in multiple sclerosis-from diagnosis to prediction of disease activity. Wien Med Wochenschr (2022) 172(15-16):337–45. doi: 10.1007/s10354-022-00912-7 PMC9606042 35133530

[5] Hegen H Walde J Berek K Arrambide G Gnanapavan S Kaplan B. Cerebrospinal fluid kappa free light chains for the diagnosis of multiple sclerosis: A systematic review and meta-analysis. Mult Scler (2023) 29(2):169–81. doi: 10.1177/13524585221134213 PMC9925892 36453167

[6] Velthuis HT Knop I Stam P van den Broek M Bos HK Hol S. N Latex FLC - new monoclonal high-performance assays for the determination of free light chain kappa and lambda. Clin Chem Lab Med (2011) 49(8):1323–32. doi: 10.1515/CCLM.2011.624 21663464

[7] Bradwell AR Carr-Smith HD Mead GP Tang LX Showell PJ Drayson MT. Highly sensitive, automated immunoassay for immunoglobulin free light chains in serum and urine. Clin Chem (2001) 47(4):673–80. doi: 10.1093/clinchem/47.4.673 11274017

[8] Brennan RL Prediger DJ. Coefficient kappa: Some uses, misuses, and alternatives. Educ psychol measurement (1981) 41(3):687–99. doi: 10.1177/001316448104100307

[9] Hegen H Arrambide G Gnanapavan S Kaplan B Khalil M Saadeh R. Cerebrospinal fluid kappa free light chains for the diagnosis of multiple sclerosis: A consensus statement. Mult Scler (2023) 29(2):182–95. doi: 10.1177/13524585221134217 PMC9925908 36527368

[10] Levraut M Laurent-Chabalier S Ayrignac X Bigaut K Rival M Squalli S. Kappa free light chain biomarkers are efficient for the diagnosis of multiple sclerosis: A large multicenter cohort study. Neurol Neuroimmunol Neuroinflamm (2023) 10(1). doi: 10.1212/NXI.0000000000200049 PMC9663206 36376096

